# Development and validation of an immune‐related prognostic signature in lung adenocarcinoma

**DOI:** 10.1002/cam4.3240

**Published:** 2020-06-26

**Authors:** Sijin Sun, Wei Guo, Zhen Wang, Xin Wang, Guochao Zhang, Hao Zhang, Renda Li, Yibo Gao, Bin Qiu, Fengwei Tan, Yushun Gao, Qi Xue, Shugeng Gao, Jie He

**Affiliations:** ^1^ Department of Thoracic Surgery National Cancer Center/National Clinical Research Center for Cancer/Cancer Hospital Chinese Academy of Medical Sciences and Peking Union Medical College Chaoyang District Beijing China

**Keywords:** biomarker, infiltrated immune cell, lung adenocarcinoma, prognostic signature, tumor immune microenvironment

## Abstract

**Background:**

Lung adenocarcinomas (LUAD) is the most common histological subtype of lung cancers. Tumor immune microenvironment (TIME) is involved in tumorigeneses, progressions, and metastases. This study is aimed to develop a robust immune‐related signature of LUAD.

**Methods:**

A total of 1774 LUAD cases sourced from public databases were included in this study. Immune scores were calculated through ESTIMATE algorithm and weighted gene co‐expression network analysis (WGCNA) was applied to identify immune‐related genes. Stability selections and Lasso COX regressions were implemented to construct prognostic signatures. Validations and comparisons with other immune‐related signatures were conducted in independent Gene Expression Omnibus (GEO) cohorts. Abundant infiltrated immune cells and pathway enrichment analyses were carried out, respectively, through ImmuCellAI and gene set enrichment analysis (GSEA).

**Results:**

In Cancer Genome Atlas (TCGA) LUAD cohorts, immune scores of higher levels were significantly associated with better prognoses (*P* < .05). Yellow (n = 270) and Blue (n = 764) colored genes were selected as immune‐related genes, and after univariate Cox regression analysis (*P* < .005), a total of 133 genes were screened out for subsequent model constructions. A four‐gene signature (ARNTL2, ECT2, PPIA, and TUBA4A) named IPSLUAD was developed through stability selection and Lasso COX regression. It was suggested by multivariate and subgroup analyses that IPSLUAD was an independent prognostic factor. It was suggested by Kaplan‐Meier survival analysis that eight out of nine patients in high‐risk groups had significantly worse prognoses in validation data sets (*P* < .05). IPSLUAD outperformed other signatures in two independent cohorts.

**Conclusions:**

A robust immune‐related prognostic signature with great performances in multiple LUAD cohorts was developed in this study.

## Graphical abstract

An immune‐related four‐gene prognostic signature was constructed using stability selection and penalized COX regression. Functional analysis suggested this signature was closely associated with innate immunity component. Our signature may server as a promising prognostic biomarker for LUAD in the future.

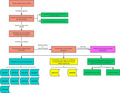

## 1 INTRODUCTION

According to estimates of cancer incidence in GLOBOCAN 2018, lung cancers remain a leading cause of cancer‐related deaths, which are cancers with the highest death incidence worldwide.^1^ Approximately, 85% of lung cancers are NSCLCs, which can be further divided into three subtypes: large cell carcinomas, squamous cell carcinomas, and lung adenocarcinomas (LUAD).^2^, ^3^ In most countries, LUAD is the most common histological subtype, which is a result of an increasing number of nonsmokers.^4^ With rapid evolvements of precision medicines, novel therapeutic strategies, especially immunotherapies, have been proposed to improve clinical outcomes of LUAD patients.^5^, ^6^ However, only a fraction of patients was benefited from immunotherapies, leaving urgencies of finding potential biomarkers for efficient and prognostic predictions.

Cytotoxic T‐lymphocyte‐associated Antigen 4 (CTLA‐4) and Programmed Death 1 (PD‐1) immune checkpoints start a new paradigm shift in the field of immunotherapies.^7^ Mechanisms of immune checkpoint inhibitors (ICIs) targeting at these molecules are to relieve certain inhibitory pathways and thus, boosting the immune system to generate antitumor activities.^8^ Accordingly, efficacies of ICIs are considered strongly associated with hosts’ immune systems and tumor immune microenvironments (TIME). Crosstalk between cancer cells and TIME was sophisticated comprising both protumorigenic and antitumorigenic manners.^9^ A T cell inflamed TIME induced by a CDK4/6 Inhibitor was shown to enhance efficacies of ICIs in vitro.^10^ Therefore, in‐depth understanding of TIME can assist identifications of novel predictive biomarkers and developments of new therapeutic strategies.

With diminishing costs, high‐throughput sequencing has emerged as a commonplace technology in the field of molecular biology. Computational algorithms, including Cibersort,^11^ Timer,^12^ and ImmuCellAI,^13^ have also been developed for assessments on abundance of infiltrated immune cells based on gene expression profiles. Altogether, these techniques provided valid and economical methods for providing detailed TIME profiles. Recently, several studies have been devoted to constructing immune‐related signatures in LUAD^14^, ^15^ and.^16^ Gene selections in their studies were based on prior knowledge sourced from external databases or differentially expressed genes (DEGs). Models based on these criteria might exclude immune‐related genes that had not yet been confirmed or prognostic genes without different expressions. In addition, models containing too many genes limited feasibilities of their clinical applications. Therefore, there is an urgent need to construct a robust and simple immune‐related prognostic signature.

In this study, immune scores of each case related to TCGA‐LUAD were first calculated through ESTIMATE algorithm, then, weighted gene co‐expression network analysis (WGCNA) was applied to identify immune‐related modules. After that, a three‐gene prognostic model was constructed based on stability selection and Lasso COX regression, whose effectiveness was further evaluated based on nine independent data sets and compared with other previously reported immune‐related signatures. Finally, it was suggested by infiltrated immune cells and pathway enrichment analysis that our prognostic signature was closely related to components of innate immunities. In conclusion, we constructed a robust immune‐related signature based on transcriptomics.

## 2 MATERIALS AND METHODS

### 2.1 Data acquisition and preprocessing

RNA‐seq mRNA expression profiles and clinical information of TCGA‐LUAD cohorts were downloaded from the Cancer Genome Atlas (TCGA) Genomic Data Commons Data Portal (https://portal.gdc.cancer.gov/). Microarray‐based data about expressions, which contained nine Gene Expression Omnibus (GEO) data sets, was obtained from official website of GEO (https://www.ncbi.nlm.nih.gov/geo/) via GEOquery R package.^17^ Only patients with pathologically confirmed LUAD and complete information about transcriptomics overall survivals (OS) could be considered. Finally, a total of 1774 LUAD cases (TCGA:504, GEO:1270) sourced from TCGA and GEO databases were included in our study.

For TCGA‐LUAD cohorts, paired‐normal samples were first removed according to their TCGA barcodes. Then, Fragments per Kilobase Million (FPKM) values were transformed into Transcripts Per Million (TPM). Average expression value of multiple samples corresponding to the same patients was calculated for further analyses.

For GEO data sets, probe IDs were converted to gene symbols according to platform annotation files. Normalized expression values were logarithmically transformed and scaled before being used in model validations. Average value of genes with multiple probes was used as their expression value.

### 2.2 Evaluating tumor microenvironment in TCGA‐LUAD cohort

ESTIMATE is a computerized algorithm which can be used to infer infiltration levels of stromal and immune cells in tumor tissues based on expression profiles.^18^ Stromal, immune, and ESTIMATE scores of each sample in TCGA‐LUAD cohorts were downloaded from the official website (https://bioinformatics.mdanderson.org/estimate/). Survival and association analyses were performed between tumor stages and these scores.

### 2.3 Construction of weighted gene co‐expression networks and identification of immune‐related modules

WGCNA is a systematic biological method developed by Langfelder et al^19^ It can be used to cluster highly correlated genes into modules and relative modules to phenotypes of interest. The WGCNA package of R software (http://www.rproject.org/) was used for network developments and visualizations. In network construction processes, soft thresholding power β was chosen as the lowest power with which fit index of scale‐free topology reached 0.90. The minimum module size was set as 30. After clustering, modules were displayed together through a dendrogram with colored assignments. To identify immune‐related modules, a module‐trait relationship heatmap was drawn with correlated coefficients and *P*‐value. Two modules, which were positively and negatively related to immune scores, respectively, were selected after comprehensive considerations on both module size (n > 100) and significances of the association.

### 2.4 Development and validation of the immune‐related prognostic signature for lung adenocarcinomas (IPSLUAD)

A prognostic signature was developed in the following steps. First, expression values of 1057 genes were extracted from two selected modules of TCGA‐LUAD cohort. Univariate Cox proportional hazard regressions were performed on these genes to identify factors related to prognoses (*P* < .05). Then, stability selection method was implemented to further narrow the scope through R package c060.^20^ The logic of stability selection is to induce additional randomizations and find out which features are consistently important in every subsampling step. Finally, a Lasso‐penalized Cox model was applied to construct a prognostic model based on genes selected in previous steps through R package glmnet. A 10‐fold cross validation was performed to determine the optimal value of Lasso penalty parameter. Coefficients of each gene in the model were determined by Lasso‐penalized Cox model. The formula is:

$$\sum_{k=0}^{n}\beta_{k}G_{k},$$

where *β_k_* is coefficient of Gene *k*, and *G_k_* is the normalized expression value of Gene *k*. Survival analyses were performed in TCGA‐LUAD cohort using Kaplan‐Meier estimator and multivariate Cox regression. Cutoff value was set as median of IPSLUAD score.

To further validate prognostic value of IPSLUAD, Kaplan‐Meier survival analysis and Cox regression were performed in nine independent GEO LUAD data sets (GSE3141, GSE13213, GSE14814, GSE29016, GSE30219, GSE31210, GSE37745, GSE50081, and GSE68465)^21^, ^22^, ^23^, ^24^, ^25^, ^26^, ^27^, ^28^ and,^29^ where cutoff value was set as median of IPSLUAD score. Gene expression values were normalized and scaled before validations. Area under the curve (AUC) of each data set was calculated for detailed evaluations.

### 2.5 Estimating immune cell infiltrations between high‐risk and low‐risk groups stratified by IPSLUAD

Infiltrated immune cells, particularly T cells, play indispensable roles in tumor immunotherapies. To compare infiltrated immune cells in samples with different IPSLUAD scores, ImmuCellAI was adopted to calculate the abundance of 24 immune cell types including 18 T‐cell subsets. For cells with a median abundance that was higher than 0.1 and significant differences among groups (*P* < .05), association analyses between markers of these cells and IPSLUAD scores were performed to validate those differences. Cell markers were chosen according to CellMarker database.^30^

### 2.6 Functional enrichment analysis of IPSLUAD using gene set enrichment analysis (GSEA)

To further understand biologic functions of IPSLUAD, GSEA was performed on TCGA‐LUAD cohort based on GO Biological Process Ontology and KEGG gene sets.^31^ False discovery rate (FDR) was introduced as a control of Type I errors, and FDRs lower than 0.05 were considered significant. All the analyses were implemented through GSEA software (version 4.0.2).

### 2.7 Comparison between IPSLUAD and other existing immune‐related signatures

In order to further evaluate prediction accuracies of IPSLUAD, we compared IPSLUAD with other immune‐related signatures ranging from three to thirty genes (Table S3). Two large independent cohorts (n > 200, GSE31210, GSE68465) were employed for comparisons.^24^, ^29^ Risk scores of each signature were calculated based on normalized expression values and coefficients provided by original articles. Two‐year and five‐year AUCs were calculated and compared based on two data sets.

### 2.8 Statistical analysis

Continuous variables were summarized through mean and standard deviations and compared through Wilcoxon test. Categorized variables were presented by frequency (n) and proportion (%), and then compared through ANOVA. Both Cox proportional hazard model and Log‐rank test were applied to survival analyses, all of which were performed through R software (Version 3.6.3, The R Foundation for Statistical Computing). *P* values were two‐side and were considered to be statistically significant if they were lower than .05.

## 3 RESULTS

### 3.1 Association between ESTIMATE‐calculated scores and clinicopathological indicators

**Figure 1:**
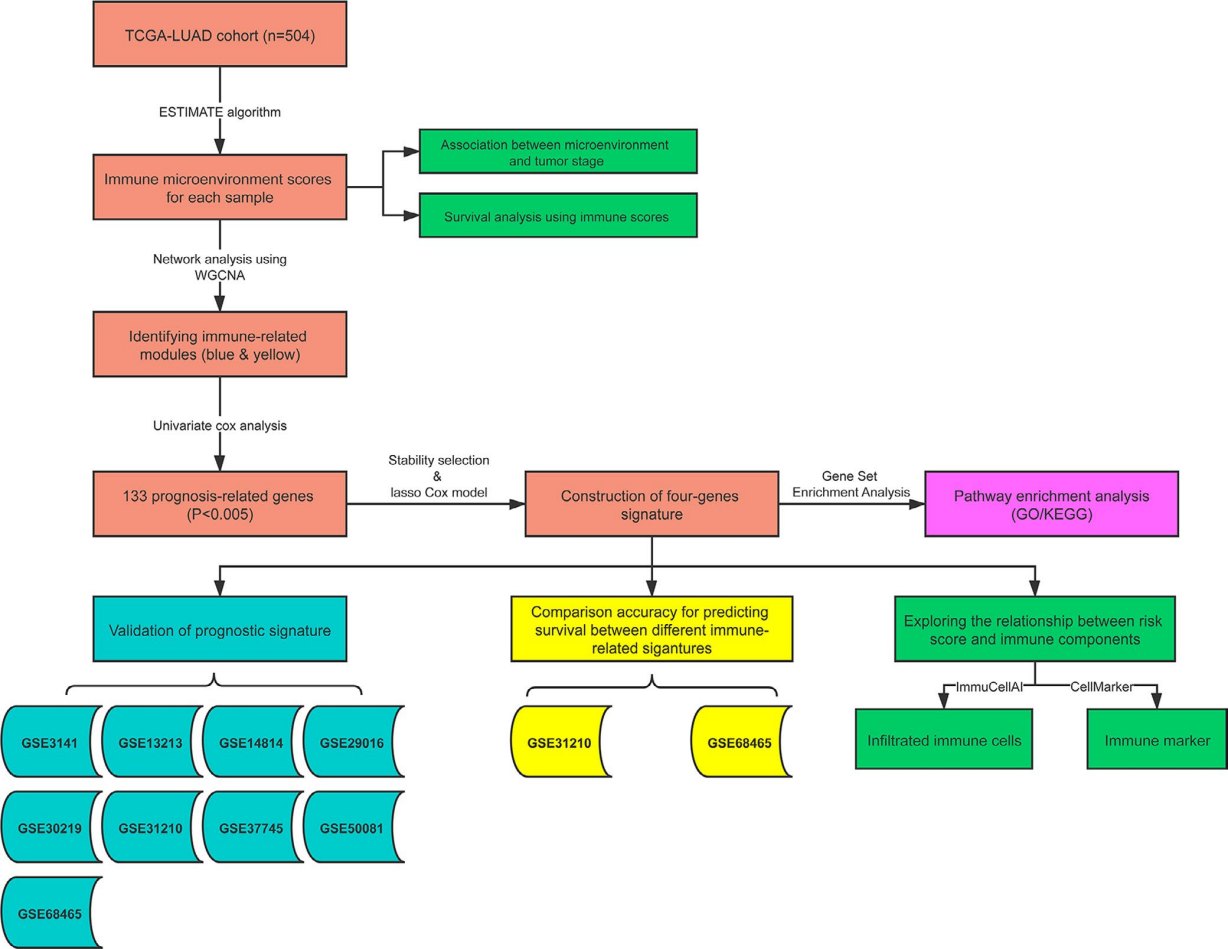
Flowchart of developments and validations of IPSLUAD

**Figure 2:**
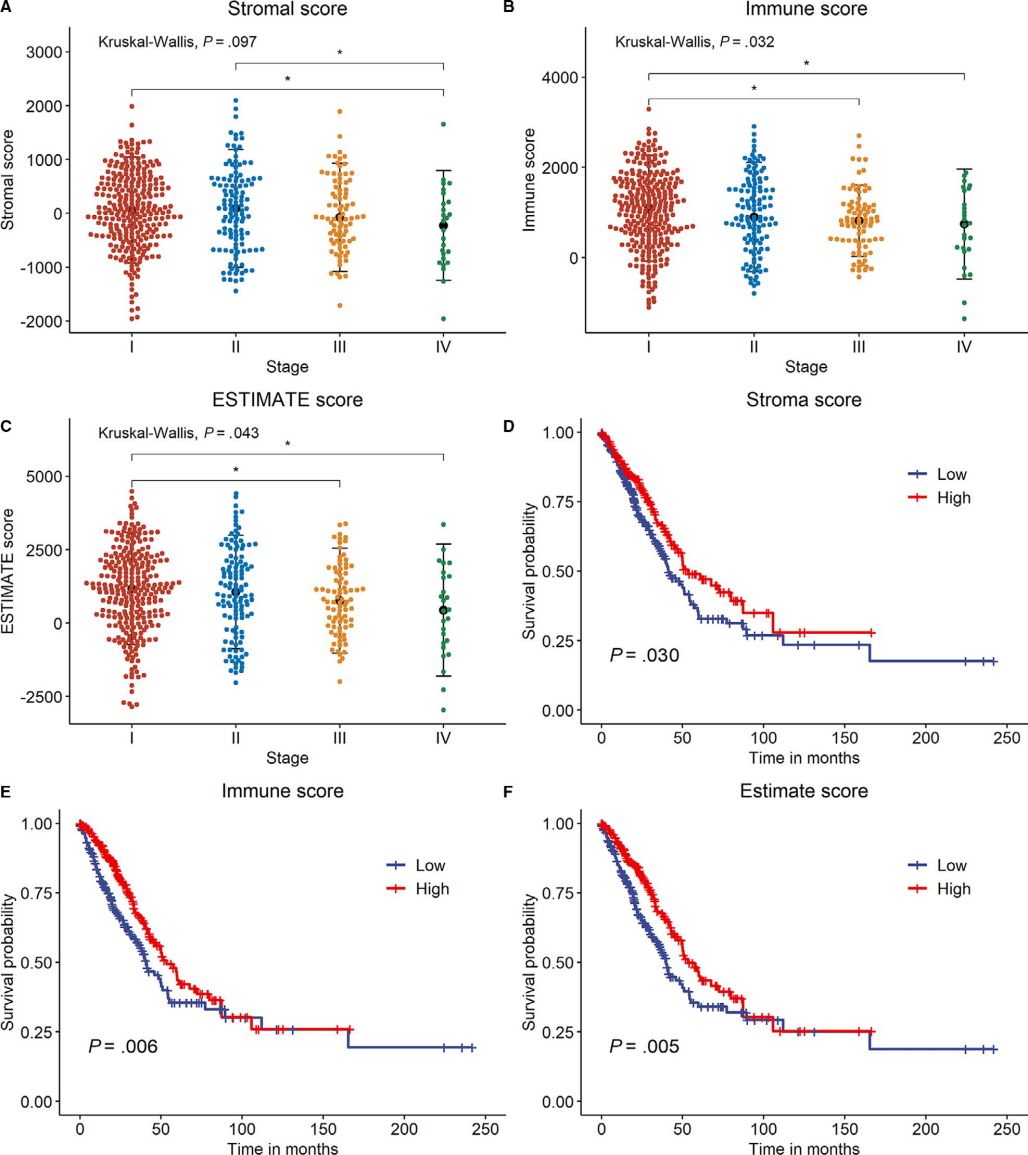
Associations between immune/stromal/ESTIMATE scores and clinicopathological indicators. A‐C, Differences among stromal/immune/ESTIMATE scores in different pathologic stages. Asterisk (*) indicated a significant difference between two groups (*P* < .05). D‐F, Survival analyses of stromal/immune/ESTIMATE scores through Kaplan‐Meier curve with log‐rank test

The whole processes of signature constructions and data analyses were shown in Figure 1. After excluding 13 cases with incomplete stages or survival information, 504 cases in TCGA‐LUAD cohort were eligible for analyses. In each sample, stromal, immune and ESTIMATE scores were obtained, which, respectively, ranged from −1779.3 to 2106.9, −932.6 to 3237.6, and −2338.0 to 4907.6. In difference comparisons, immune and ESTIMATE scores were significantly associated with pathologic stages (Figure 2A‐C, *P* < .05). The lowest stromal, immune, and ESTIMATE scores were all found in cases of Stage IV, which was the most progressive. In addition, cases with higher stromal, immune or ESTIMATE scores tended to have a better prognosis than those with scores of lower levels (Figure 2D‐F).

### 3.2 Construction of co‐expression modules and identification of modules associated with immune scores

**Figure 3:**
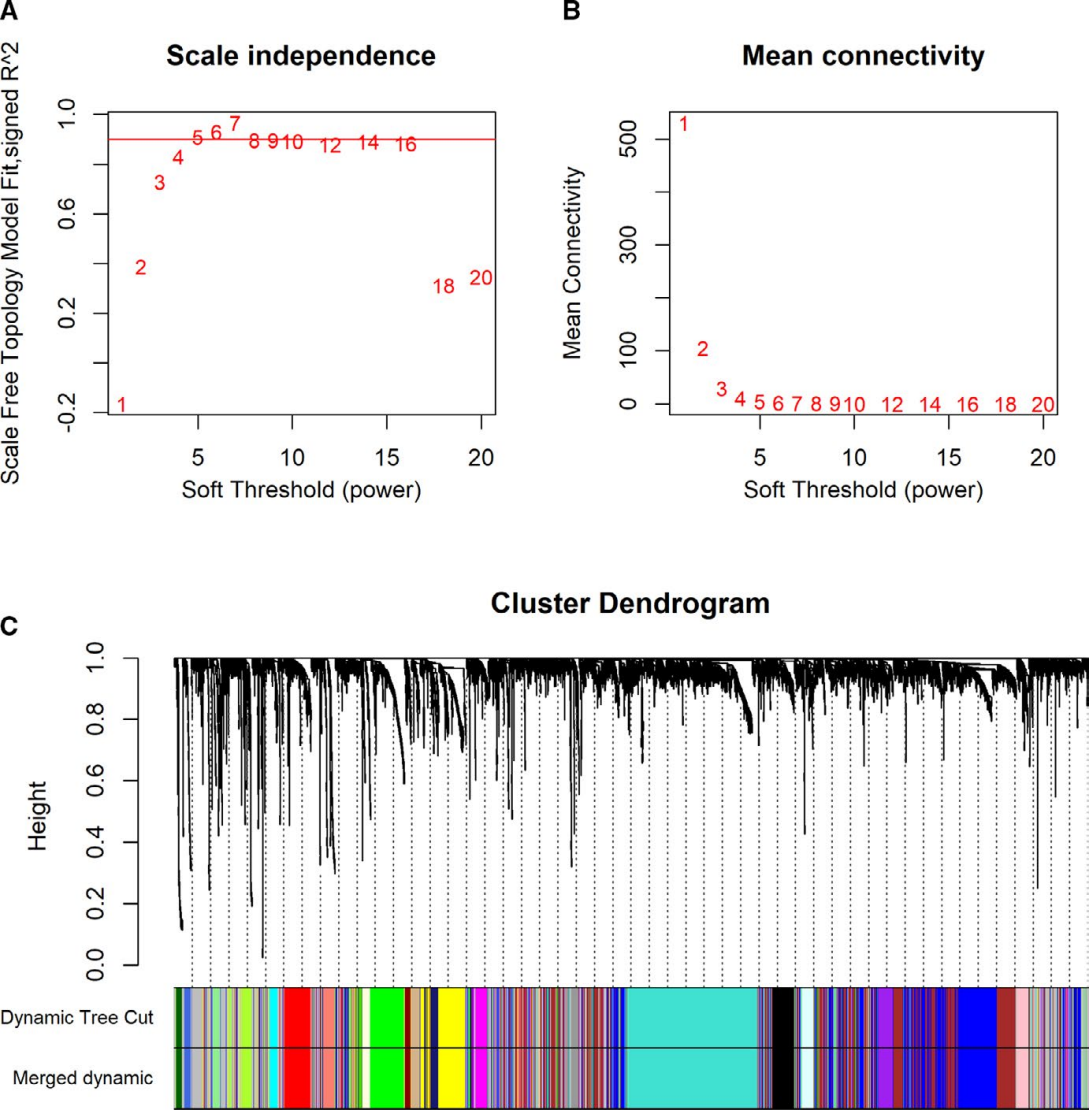
Network constructions and module detections of LUAD. A and B, Analyses of network topologies for various soft‐thresholding powers through scale‐free fit index (A) and mean connectivity (B). C, Clustering dendrogram of genes based on topological overlapping. Different colors were assigned to corresponding modules. A total of 23 modules were identified

Network constructions and module detections were followed by a step‐by‐step approach. First, soft threshold power 5 was chosen to calculate adjacencies since it was the lowest power with which fit index of scale‐free topologies reached 0.90 (Figure 3A and B). Then, module identifications were performed through a dynamic tree cut with a deepSplit parameter set as 2. After merging similar modules, a total of 23 modules were identified and clustering dendrograms were presented (Figure 3C). A heatmap was drawn to shown correlated modules, in which red color represented positive correlations and blue color represented negative ones (Figure S2A). To identify immune‐related modules, module‐trait association plots were presented (Figure S1). Results demonstrated that the top three modules with the greatest positive associations were colored with Yellow, Blue, and Tan, while the top three ones with the greatest negative associations were colored with Black, Purple, and Blue. Among these modules, genes colored with Blue (n = 764) and Yellow (n = 270) were selected in module constructions based on module sizes (n > 200) and association significances. Finally, association analyses of Gene Significances (GS) and module membership (MM) were performed on Blue and Yellow modules. GSs of all the above three scores were significantly (*P* < .05) associated with MM in these two modules (Figure S2B‐G).

### 3.3 Construction of IPSLUAD in TCGA‐LUAD cohort

A total of 1034 genes were selected through previous steps (Table S1). To explore genes significantly associated with OS of patients with LUAD, univariate Cox regression analysis was performed and genes with *P* values of lower than .005 were chosen. 133 genes were screened out and selected for constructions of subsequent models (Table S2). Stability selection was first implemented to identify important features, and estimated sets of stable features comprised four genes, namely Aryl Hydrocarbon Receptor Nuclear Translocator Like 2 (ARNTL2), Epithelial Cell Transforming 2 (ECT2), Peptidylprolyl Isomerase A (PPIA), and Tubulin Alpha 4a (TUBA4A) (Figure S3A). Then, the optimal value of Lasso penalty parameter λ was determined as 0.0048 through 10‐fold cross validation (Figure S3B‐C). λ was then substituted into the model to generate coefficients of each gene. The final IPSLUAD was calculated as follows: (0.2319 × EXP_ARNTL2_) + (0.1595 × EXP_ECT2_) + (0.1611 × EXP_PPIA_) + (0.1286 × EXP_TUBA4A_).

**Figure 4:**
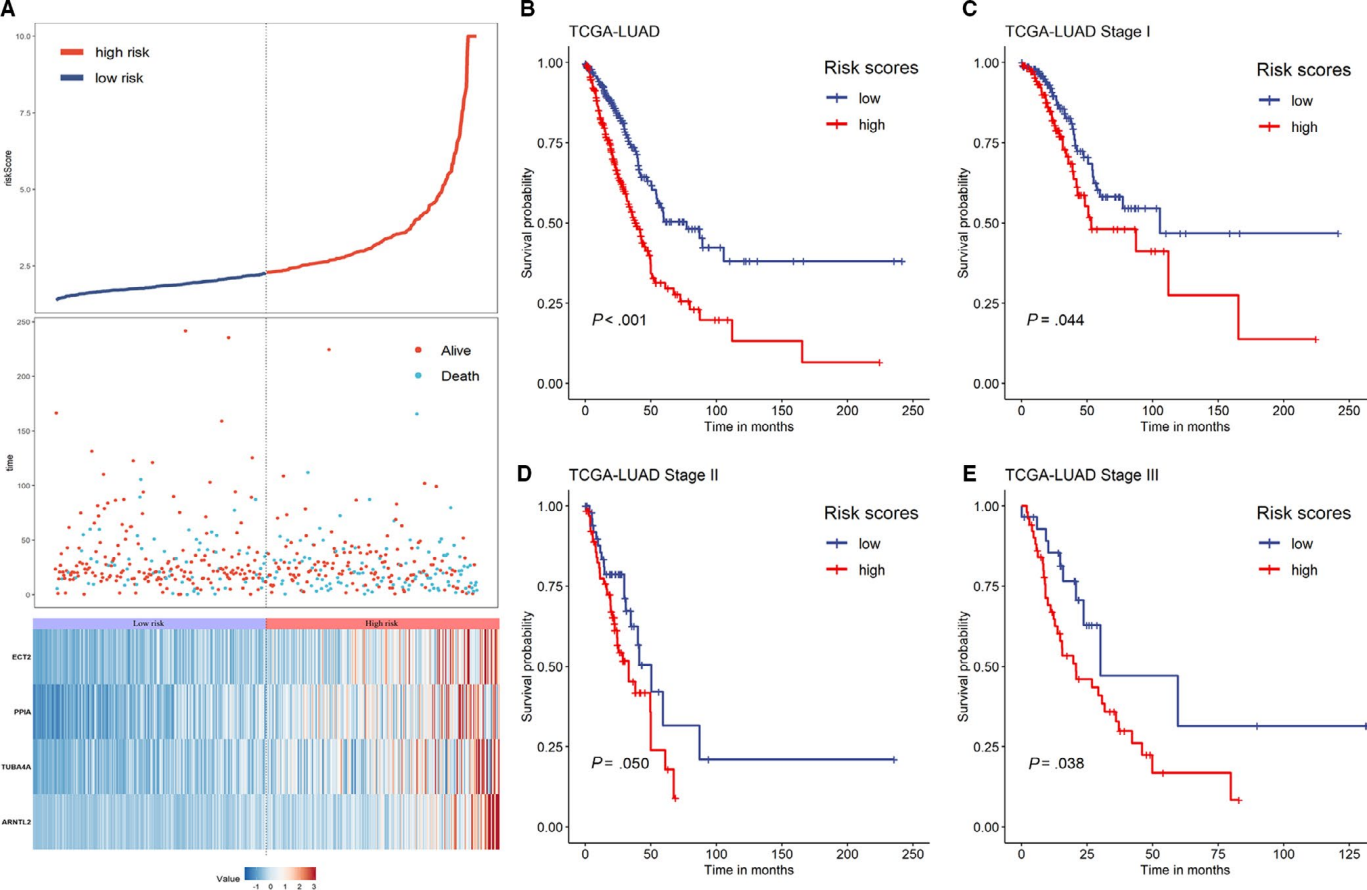
Prediction performances of IPSLUAD in TCGA‐LUAD cohort. A, Distributions of risk scores (top), survival statuses of patients in low‐risk and high‐risk groups (middle), and four‐gene expression profiles of each patient (bottom). B‐E, Kaplan‐Meier curves of OS between low‐risk and high‐risk groups based on whole‐TCGA cohort (B), Stage I (C), Stage II (D), and Stage III subgroup (E)

IPSLUAD of each patient in TCGA‐LUAD was calculated, and high‐/low‐risk groups were divided with median IPSLUAD as cutoff. Distributions of risk scores, survival statuses, and four‐gene expression profiles were shown in Figure 4A. It was demonstrated by association analyses that factors including genders, T Stage, N Stage, pathological stages, and survival statuses were significantly correlated (*P* < .05) with different IPSLUAD groups (Table 1). Patients in high‐risk group had significantly worse prognoses than those in low‐risk group (Log‐rank *P* < .05) (Figure 4B). In subgroup analyses, a significantly shorter OS was shown in high‐risk group than low‐risk group in Stage I and III (Log‐rank *P* < .05) (Figure 4C and E). A similar trend was found among patients in Stage II, although there were no statistical significances (Log‐rank *P* = .050) (Figure 4D). Univariate Cox regressions showed that T Stage, lymph node involvements, distant metastases, pathological stage, and IPSLUAD were significantly associated with OS (Table 2). Further, multivariate Cox regressions demonstrated that T Stage, lymph node involvements, and IPSLUAD were independent prognostic factors (Table 2).

**Table 1:** Correlations between IPSLUAD and clinicopathological parameters of 465 patients in TCGA‐LUAD cohort

Category	Cases, 465 (100%)	IPSLUAD		*P* value
		Low (n = 232)	High (n = 233)	
Age, mean (SD)		66.1 (9.5)	64.3 (10.5)	0.060
Gender				
Male	211 (45.4%)	90 (38.8%)	121 (51.9%)	**0.005**
Female	254 (54.6%)	142 (61.2%)	112 (48.1%)	
Smoking				
Ever	400 (86.0%)	199(85.8%)	201 (86.3%)	0.985
Never	65 (14.0%)	33 (14.2%)	32 (13.7%)	
T stage				
T1	161 (34.6%)	98 (42.2%)	63 (27.0%)	**0.006**
T2	245 (52.7%)	110 (47.4%)	135 (57.9%)	
T3	44 (9.4%)	17 (7.3%)	27 (11.6%)	
T4	15 (3.2%)	7 (3.0%)	8 (3.4%)	
N stage				
N0	314 (67.5%)	175 (75.4%)	139 (59.7%)	**0.004**
N1	84 (18.1%)	33 (14.2%)	51 (21.9%)	
N2	65 (14.0%)	23 (9.9%)	42 (18.0%)	
N3	2 (0.4%)	1 (0.4%)	1 (0.4%)	
M stage				
M0	444 (95.5%)	225 (97.0%)	219 (94.0%)	0.184
M1	21 (4.5%)	7 (3.0%)	14 (6.0%)	
TNM stage				
I	257 (55.3%)	146 (62.9%)	111 (47.6%)	**0.005**
II	112 (24.1%)	51 (22.0%)	61 (26.2%)	
III	75 (16.1%)	28 (12.1%)	47 (20.2%)	
IV	21 (4.5%)	7 (3.0%)	14 (6.0%)	
Status				
Survival	300 (64.5%)	172	128	**<0.001**
Death	165 (35.5%)	60	105	

Significant values of *P* < 0.05 is indicated in bold.

**Table 2:** Univariate analysis and multivariate analysis of risk factors for prognosis in TCGA‐LUAD cohort

	Univariate analysis			Multivariate analysis		
	*P* value	HR	95% CI	*P* value	HR	95% CI
Age						
≤66 years	0.091	1.302	0.959‐1.769			
>66 years						
Gender						
Female	0.353	1.156	0.851‐1.569			
Male						
Smoking						
Never	0.985	0.996	0.640‐1.549			
Ever						
T stage						
I‐II	**<0.001**	2.313	1.557‐3.436	**0.003**	1.920	1.246‐2.959
III‐IV						
Lymph node metastasis						
Negative	**<0.001**	2.507	1.843‐3.411	**<0.001**	2.088	1.429‐3.050
Positive						
Distant metastasis						
No	**0.018**	1.188	1.030‐1.369	0.283	1.091	0.931‐1.278
Yes						
TNM stage						
I‐II	**<0.001**	2.343	1.687‐3.254	0.825	1.054	0.660‐1.683
III‐IV						
IPSLUAD						
Low‐risk	**<0.001**	2.178	1.582‐2.999	**<0.001**	1.875	1.351‐2.602
High‐risk						

Significant values of *P* < 0.05 is indicated in bold.

### 3.4 Validation of IPSLUAD in nine GEO data sets

**Figure 5:**
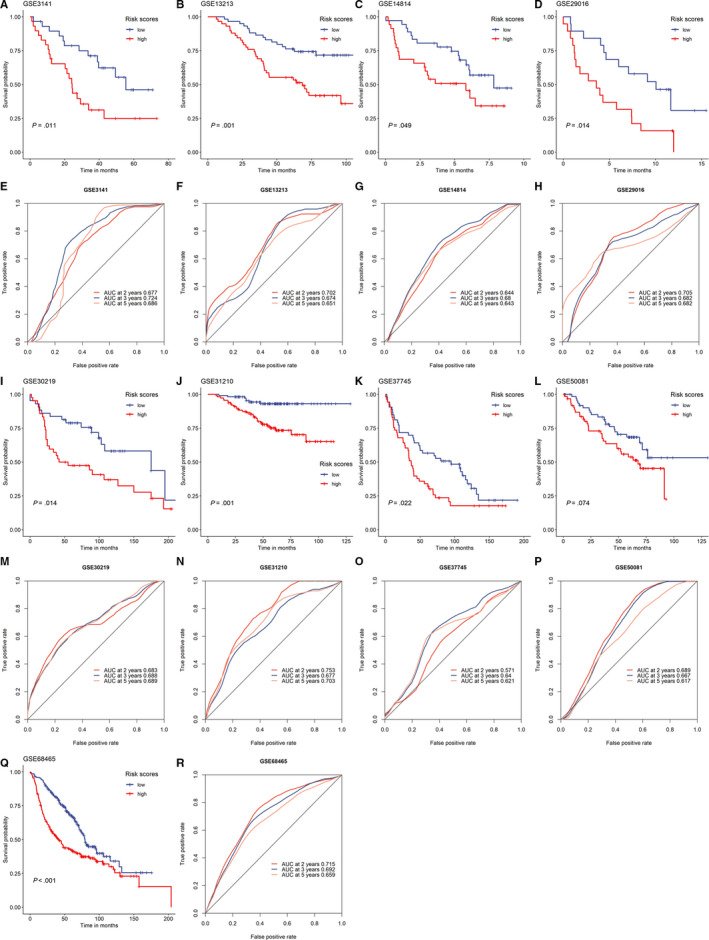
Prediction performances of IPSLUAD in validating data sets. Kaplan‐Meier survival curves of overall survivals in (A) GSE3141, (B) GSE13213, (C) GSE14814, (D) GSE29016, (I) GSE30219, (J) GSE31210, (K) GSE37745, (L) GSE50081, (Q) GSE68465. Receiver operating curve (ROC) analysis of IPSLUAD in (E) GSE3141, (F) GSE13213, (G) GSE14814, (H) GSE29016, (M) GSE30219, (N) GSE31210, (O) GSE37745, (P) GSE50081, (R) GSE68465

Summaries of the nine GEO data sets were shown in Table 3. In each data set, patients were stratified into high‐/low‐risk groups according to the median of IPSLUAD. It was suggested by Kaplan‐Meier survival analyses that patients in high‐risk group had significantly worse prognoses in eight out of nine data sets (88.9%) (Figure 5A‐D, I‐K, Q). The same trend was also observed in IPSLUAD of one remaining data set, though it was not statistically significant (Figure 5L). AUC was also calculated to evaluate abilities of IPSLUAD, which was ranged from 0.617 to 0.753 in the nine data sets (Figure 5E‐H, M‐P, R). Importantly, there were five data sets (GSE3141, GSE13213, GSE29016, GSE31210, and GSE68465) with AUC values that were higher than 0.7.

**Table 3:** Characteristics of GEO data sets used in this study

Author	GEO Accession	Year of publication	Platform	Number of patients	Events
Nevins et al	GSE3141	2006	GPL570 [HG‐U133_Plus_2]	58	32
Tomida et al	GSE13213	2009	GPL6480	117	49
Tsao et al	GSE14814	2010	GPL96 [HG‐U133A]	71	35
Staaf et al	GSE29016	2012	GPL6947	38	28
Rousseaux et al	GSE30219	2013	GPL570 [HG‐U133_Plus_2]	85	45
Okayama et al	GSE31210	2011	GPL570 [HG‐U133_Plus_2]	226	35
Botling et al	GSE37745	2012	GPL570 [HG‐U133_Plus_2]	106	77
Pintilie et al	GSE50081	2013	GPL570 [HG‐U133_Plus_2]	127	51
Heiskanen et al	GSE68465	2015	GPL96 [HG‐U133A]	442	236

### 3.5 Comparisons between IPSLUAD and other existing immune‐related prognostic signatures

**Figure 6:**
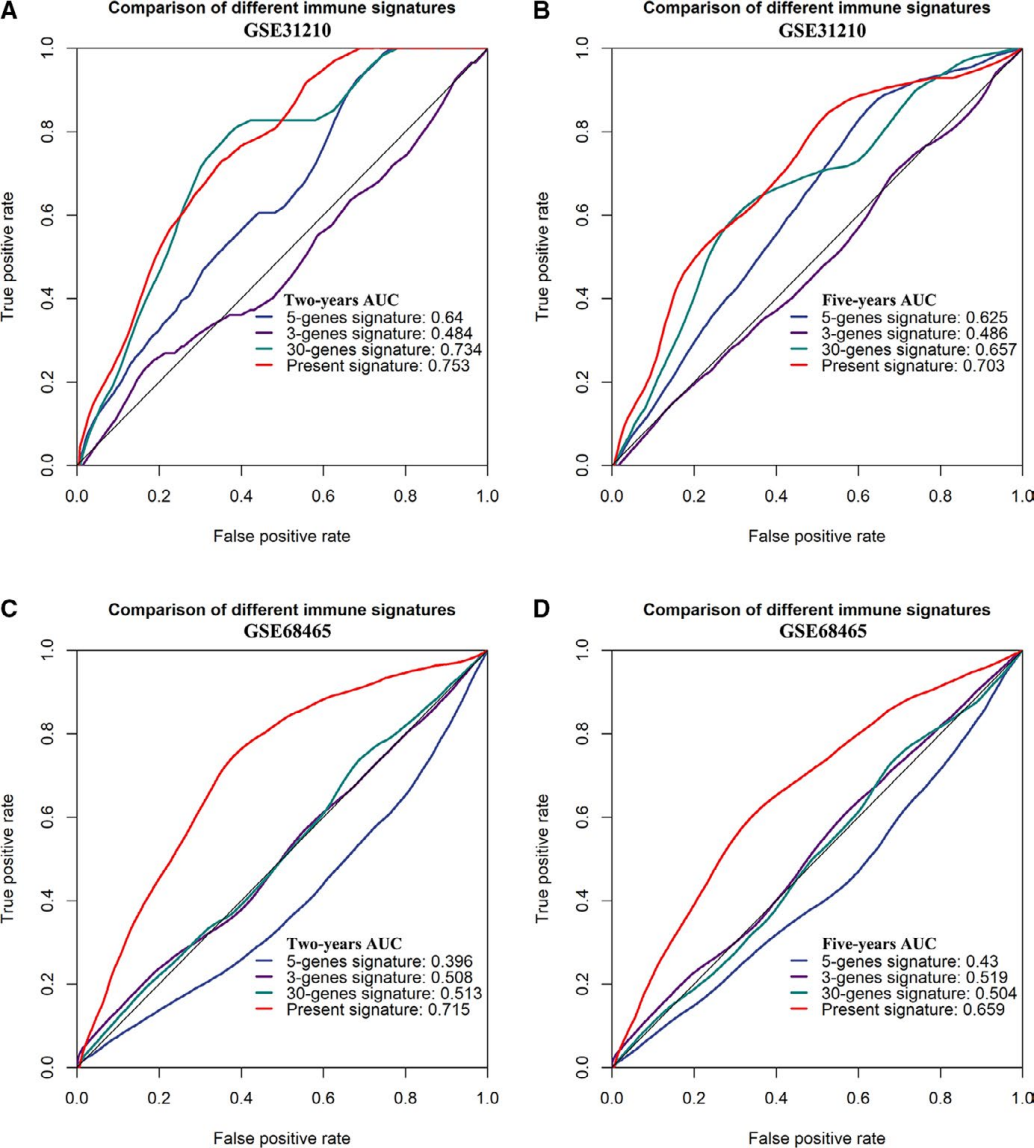
Comparisons of IPSLUAD with other published immune‐related models in GSE31210 and GSE68465 data sets. A, Two‐year ROC in GSE31210. B, Five‐year ROC in GSE31210. C, Two‐year ROC in GSE68465. D, Five‐year ROC in GSE68‐465

Characteristics of other immune‐related signatures used in this study were summarized in Table 4. Two largest GEO cohorts (GSE31210, GSE68465) with sample sizes higher than 200 were used in model comparisons. GSE31210 was an early‐stage cohort: all patients were in Stage I or II, while GSE68465 was a multi‐site cohort comprising both early‐stage patients and those in more advanced stages. For all comparisons in these two cohorts, IPSLUAD outperformed other signatures with superior predictive performances according to AUC values (0.75 vs 0.64‐0.73; 0.70 vs 0.49‐0.66; 0.72 vs 0.40‐0.51; 0.66 vs 0.43‐0.52) (Figure 6A‐D).

**Table 4:** Summary of existing immune‐related prognostic signatures

Study	Number of genes	Gene list
Li et al	5	SLCO4C1, ELAC1, HLF, ZNF204P, ST3GAL5
Yue et al	3	ADAM12, BTK, ERG
Song et al	30	PSMC6, LIFR, PIK3CG, CTF1, RELA, MAP3K8, HLA‐DOB, LGR4, RXRB, CD79A, ADIPOR2, CCL20, PTPN6, HSPA4, GPI, ADM, IL22RA1, ANGPTL4, XCR1, AP3B1,RFXAP, HSPA2, IL23R, PDGFB, DKK1, PAK4, PSMD2,VEGFC, SHC1, HGF

### 3.6 Estimating the composition of infiltrated immune cells

**Figure 7:**
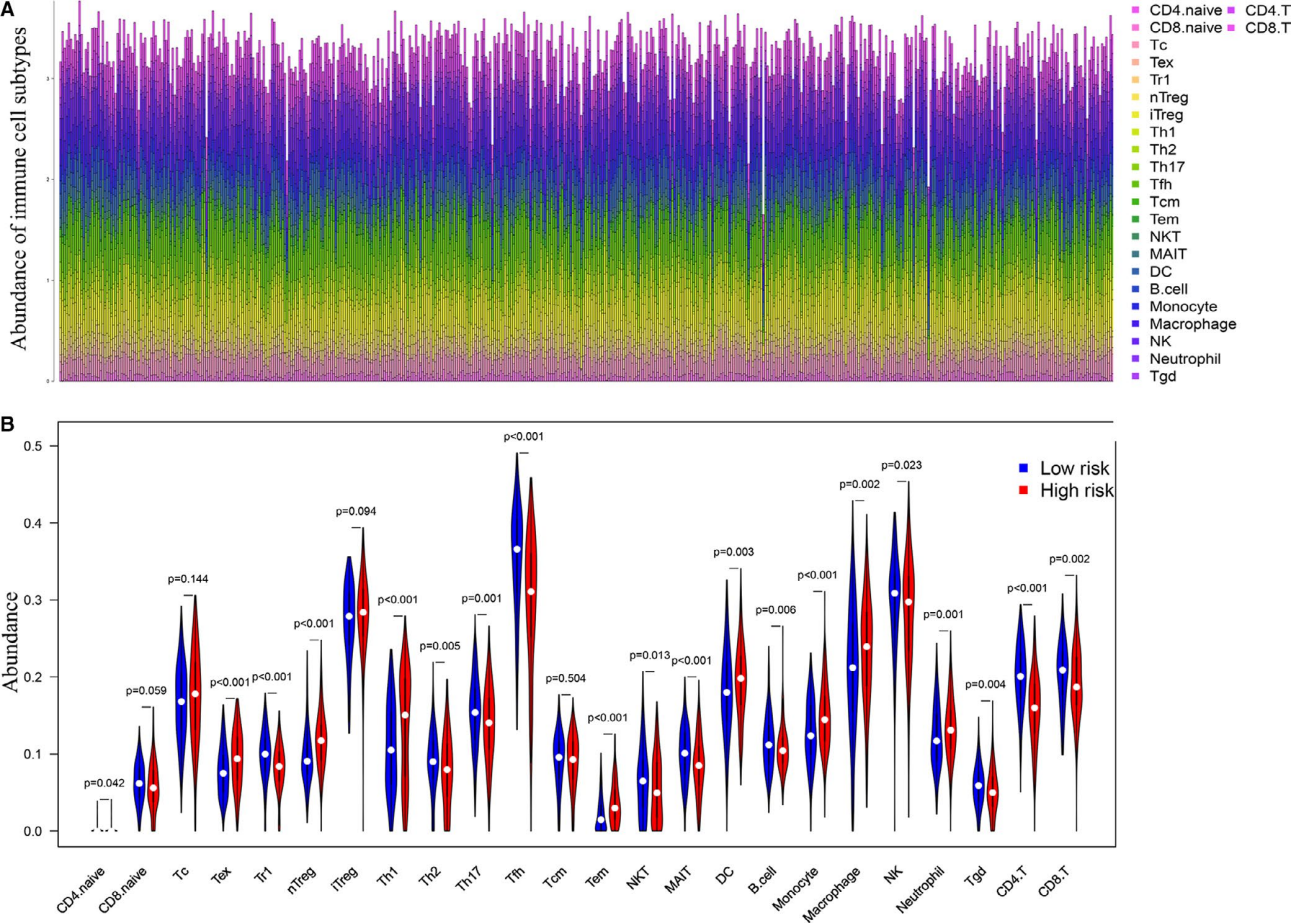
Compositions of infiltrated immune cells between low‐risk and high‐risk groups in TCGA‐LUAD cohort through ImmuCellAI. A, Abundance of 24 immune cell types in TCGA. B, Comparisons between immune cells in low‐risk and high‐risk groups in TCGA

To further characterize immune microenvironment of tumors, ImmuCellAI was employed to estimate the abundance of 24 types of immune cells in TCGA‐LUAD cohort based on RNA‐Seq data. Abundant immune cell populations with various kinds in each sample were shown in Figure 7A. Several cell types, namely CD4 naïve T cells, exhausted T cells (Tex), Type 1 regulatory T cells (Tr1), natural regulatory T (nTreg), Th1, Th2, Th17, Tfh, effector memory T (Tem) cells, natural killer T (NKT) cells, mucosal‐associated invariant T cells (MAIT), dendritic cells (DC), B cells, monocytes, macrophages, natural killer cells (NK), Neutrophil, Gamma delta T cells (Tgd), CD4 T cells, and CD8 T cells, were significantly different (*P* < .05) in high‐risk and low‐risk groups (Figure 7B).

### 3.7 Associations between IPSLUAD and immune cell markers

Different abundances of infiltrated immune cells between high‐risk and low‐risk groups were further validated through correlation analyses on gene markers and IPSLUAD. Markers significantly correlated with IPSLUAD included CD4 for CD4 T cells, CD66b and CD11b for neutrophil, CD56, CD16, and CD94 for NK cells, CD206 for M2 macrophages, CD14 for monocytes, CD19 and CD79A for B cells, CD11c and HLA‐DRA for dendritic cells, BCL6 and CD185 for Tfh, CD196, RORC, STAT3 for Th17, STAT1 for Th1 and STAT5B and HELIOS for Tregs (Table 5). It was suggested by these findings that IPSLUAD was strongly linked with innate immunities since it was closely related to several critical innate immunity‐related components (neutrophil, NK cells, and dendritic cells).

**Table 5:** Correlation analysis between IPSLUAD and immune cell markers

Marker	IPSLUAD		*P* value
	Low, median (IQR)	High, median (IQR)	
CD8^+^ T cell			
CD8A	2.39 (1.87, 2.95)	2.55 (1.82, 3.18)	0.148
CD8B	1.56 (1.08, 2.17)	1.68 (1.13, 2.35)	0.136
CD4^+^ T cell			
CD4	4.36 (3.80, 4.71)	4.19 (3.70, 4.60)	**0.035**
Neutrophil			
CD66b	0.12 (0.04, 0.44)	0.06 (0.02, 0.15)	**<0.001**
CD11b	2.79 (2.26, 3.27)	2.72 (2.06, 3.22)	**0.315**
Natural killer cell			
CD56	0.42 (0.23, 0.71)	0.30 (0.16, 0.61)	**0.001**
CD16	4.36 (3.78, 4.94)	4.61 (4.03, 5.16)	**<0.001**
CD94	0.41 (0.26, 0.68)	0.50 (0.30, 0.79)	**0.014**
M1 Macrophage			
CD86	2.81 (2.27, 3.18)	2.87 (2.41, 3.31)	0.139
CD80	1.04 (0.67, 1.35)	1.04 (0.72, 1.40)	0.533
iNOS	0.57 (0.34, 0.90)	0.65 (0.37, 0.97)	0.120
M2 Macrophage			
CD163	3.65 (3.07, 4.15)	3.75 (3.03, 4.39)	0.178
CD206	3.79 (2.92, 4.32)	3.49 (2.64, 4.17)	**0.033**
Monocyte			
CD14	4.86 (4.40, 5.26)	5.00 (4.46, 5.48)	**0.043**
CD33	1.31 (0.92, 1.72)	1.24 (0.83, 1.62)	0.156
CD172a	3.49 (3.06, 3.84)	3.56 (3.08, 3.94)	0.107
B cell			
CD19	1.65 (0.94, 2.40)	1.25 (0.78, 1.76)	**<0.001**
CD79A	4.03 (3.02, 4.78)	3.40 (2.76, 4.18)	**<0.001**
Dendritic cell			
CD11c	2.90 (2.50, 3.38)	2.77 (2.39, 3.28)	**0.035**
CD205	1.77 (1.39, 2.13)	1.82 (1.38, 2.27)	0.160
HLA‐DRA	8.17 (7.62, 8.68)	8.00 (7.32, 8.46)	**0.002**
Tfh			
BCL6	3.71 (3.46, 4.00)	3.58 (3.34, 3.90)	**0.001**
CD185	0.16 (0.06, 0.35)	0.10 (0.04, 0.20)	**<0.001**
CD278	1.27 (0.85, 1.69)	1.31 (0.90, 1.82)	0.417
Th17			
CD196	0.23 (0.10, 0.41)	0.13 (0.05, 0.28)	**<0.001**
RORC	3.39 (2.85, 3.86)	3.20 (2.52, 3.59)	**<0.001**
STAT3	4.82 (4.57, 5.03)	4.76 (4.46, 4.98)	**0.033**
Th1			
CD183	2.26 (1.70,2.79)	2.15 (1.60, 2.73)	0.472
T‐bet	1.00 (0.73, 1.48)	1.00 (0.64, 1.56)	0.410
STAT1	4.89 (4.53, 5.30)	5.35 (4.86, 5.75)	**<0.001**
STAT4	1.80 (1.51, 2.18)	1.75 (1.38, 2.25)	0.331
Treg			
FOXP3	1.96 (1.47, 2.38)	1.98 (1.57, 2.40)	0.572
STAT5B	3.46 (3.27, 3.68)	3.39 (3.13, 3.60)	**0.002**
TGFβ	4.32 (3.99, 4.60)	4.26 (3.77, 4.66)	0.619
CD304	3.77 (3.43, 4.18)	3.85 (3.35, 4.24)	0.612
HELIOS	1.71 (1.37, 1.96)	1.79 (1.44, 2.06)	**0.038**

Significant values of *P* < 0.05 is indicated in bold.

### 3.8 Pathway enrichment analysis of IPSLUAD

**Figure 8:**
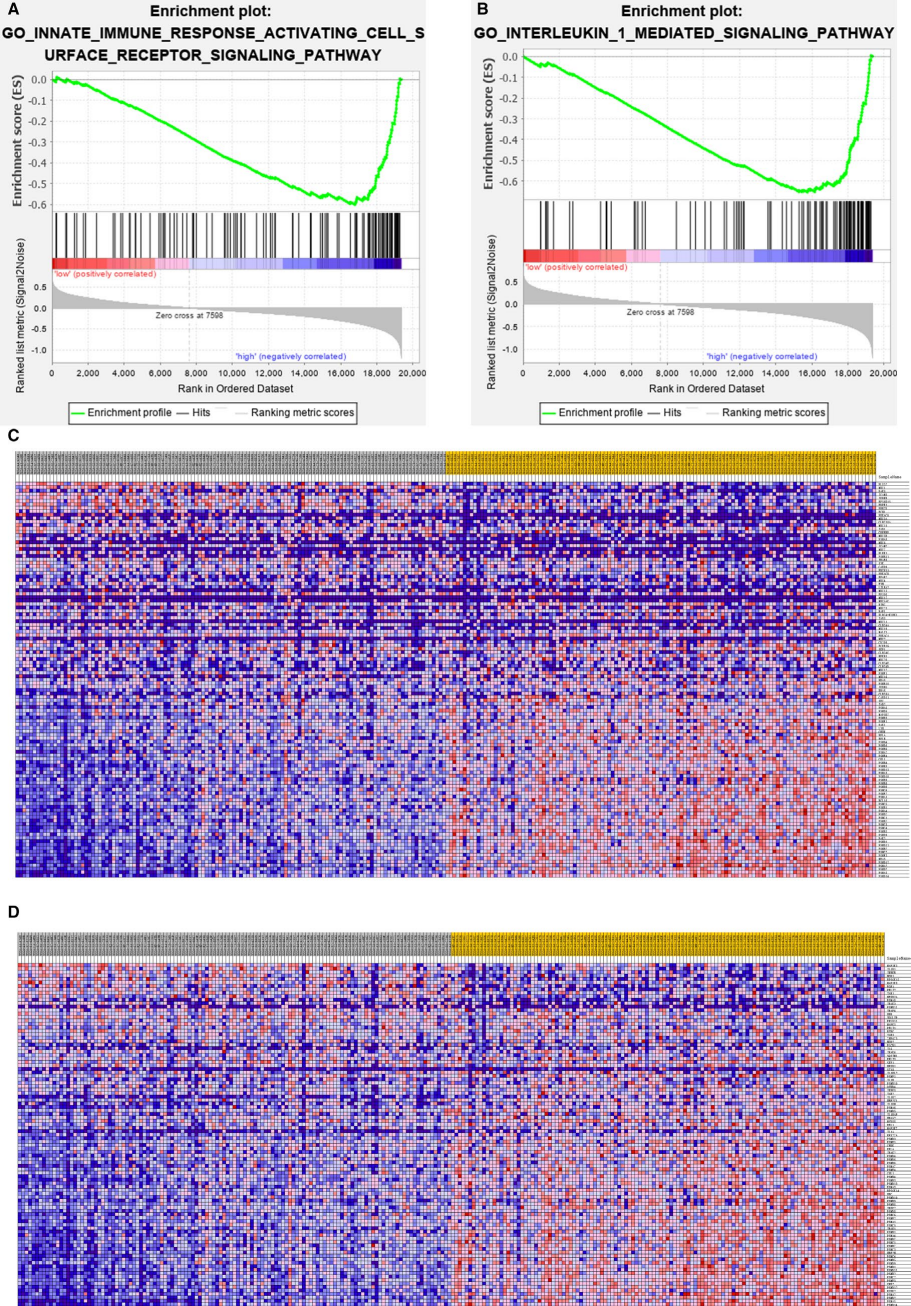
Different immune statuses between low‐risk and high‐risk groups in TCGA‐LUAD cohort. A and B, Significant enrichments of immune‐related pathways among high‐risk patients were indicated through gene set enrichment analysis (GSEA). C and D, Gene sets between low‐risk and high‐risk groups were analyzed through expression profiles of the two enrichments

To further investigate molecular functions of IPSLUAD in immune systems, a GSEA was performed based on GO Biological Process Ontology and KEGG gene sets. Two significant immune‐related pathways were identified, namely innate immune response activating cell surface receptor signaling and interleukin 1 mediated signaling pathway (Figure 8A‐D). Again, close associations between genes of IPSLUAD and innate immunities were validated through this result.

## 4 DISCUSSION

With rapid developments of molecular biology and high‐throughput sequencing, contributions of tumor microenvironment to cancer developments, progressions, and metastases have been increasingly acknowledged in recent years.^32^, ^33^ There is a complex dynamic crosstalk between tumor and non‐cancer cells including infiltrated immune cells and adjacent stroma cells.^32^ However, tumors are considered as autonomous subjects by TNM staging system, which ignores significant effects of TIME. In the present study, a novel 4‐gene immune‐related prognostic signature was developed to assist TNM staging for more accurate predictions.

Several immune‐related signatures for LUAD have been used to divide patients into different prognostic subgroups in previous researches.^14^, ^15^, ^16^ A list of genes for model constructions was extracted from public immune databases or through differential analyses. In contrast, we applied a different selection method based on co‐expression networks, which might identify immune‐related genes that had not been reported. In addition, by means of deliberately adding noises during model constructions, IPSLUAD was found to be robust with respect to OS predictions in various independent data sets. Both multivariate and subgroup analyses stratified through TNM staging suggested that IPSLUAD be used as an independent prognostic factor. Furthermore, IPSLUAD outperformed other immune‐related signatures in both early‐stage LUAD and multicenter cohorts. Therefore, IPSLUAD could not only significantly supplement traditional staging systems, but also be used as an accurate OS predictor for patients with LUAD.

Among the four genes in IPSLUAD, ECT2, and PPIA were widely studied in previous researches. ECT2 was recognized as an oncogenic gene necessary for Kras‐Trp53 lung tumorigeneses in vivo.^34^ Liu et al also reported on collaborations of ECT2 with growths of lung squamous cell carcinomas promoted by PRKCI.^35^ It was suggested by Meta‐Analysis that ECT2 was a promising prognostic factor in cancers.^36^ PPIA is a housekeeping gene involved in several cancers including NSCLC, pancreatic adenocarcinomas as well as head, and neck squamous cell carcinomas.^37^, ^38^, ^39^ PPIA was also reported to participate in tumor proliferations and invasions.^40^ However, relationships between immune cells and these genes have not been illustrated. Although little is known about roles of ARNTL2 and TUBA4A in LUAD, ARNTL2 was reported to be associated with tumor progressions and metastases in colorectal and breast cancers.^41^, ^42^ TUBA4A was found highly expressed in exosomes secreted by NSCLC cell lines in vitro.^43^ Underlying mechanisms of these two genes in LUAD still need further cellular‐level explorations.

Infiltrated immune cells constitute important parts of tumors and have been widely studied in recent years. Certain immune cell types were found to be indicative of responses to immunotherapies and OSs. Based on quantifications and locations of demonstrated T cells, the immunoscore developed by Galon et al showed superior predictive accuracies in prognoses compared to that of traditional TNM staging in colorectal carcinomas.^44^ High‐level expressions of PD1 or PD1 and CTLA4 on infiltrated CD8^+^ T cells were reported to be predictive of responses to ICIs in NSCLC.^45^ In addition, dysfunctional T cells were also considered as predictive markers of immunotherapeutic responses in melanomas.^46^ Since T cells were at the central position of antitumor immunity, a novel algorithm named ImmuCellAI was implemented to evaluate the abundance of infiltrated immune cells in TCGA cohort.^13^ Compositions of 18 T cell subsets can be calculated by ImmuCellAI, which is of superior accuracies than other frequently‐used methods including Cibersort and Timer.^11^, ^12^, ^13^ In addition to differences in multiple T cell subsets among IPSLUAD subgroups, one more intriguing finding was the significantly different pattern of innate immune systems.

In respect of innate immunity, some types of cells including DCs and macrophages are context‐specific that may exert either protumorigenic or antitumorigenic functions.^47^ The most popular “don't eat me” signals including CD47 and CD24 have been extensively studied, and drugs targeted at these molecules are currently under prudent trials.^48^, ^49^ High expressions of CD47 were also found to be associated with worse prognoses in various types of tumors.^50^ Through previous researches, innate immunity might play a more important role in the field of antitumor therapies. However, clinical significances of different innate immune statuses observed among IPSLUAD subgroups need further systematic explorations.

There were several limitations that should be stated in the present study. First, missing data and selection biases were inevitable as this was a retrospective study. Second, values of gene expressions extracted from RNA‐seqs or microarrays were all relative. Thus, absolute thresholds for stratifications among different cohorts could not be calculated. Since median cutoff values were used in each data set, accurate external validations would be needed in the future. Third, due to lack of data about immunotherapies, we were unable to investigate relationships between IPSLUADs and responses of ICIs.

## 5 CONCLUSIONS

In conclusion, a robust immune‐related prognostic signature was developed and validated in nine independent data sets. Different innate immune statuses were observed between low‐risk and high‐risk groups. This signature may serve as a promising prognostic biomarker for LUADs in the future.

## CONFLICT OF INTEREST

The authors declare that they have no competing interest.

## AUTHORS' CONTRIBUTIONS

YG, SG, and JH conceived and designed the study. ZW, XW, GZ, HZ, and RL collected the data. SS performed data analysis. SS and WG wrote the paper. BQ, FT, YG, and QX reviewed and edited the manuscript. All authors read and approved the manuscript.

## Supporting information

Fig S1

Fig S2

Fig S3

Table S1

Table S2

Table S3

## Data Availability

The data sets analysed during the current study are available in the TCGA (https://portal.gdc.cancer.gov/) and GEO repository (https://www.ncbi.nlm.nih.gov/geo/).
