# Supplementary figures and images for: Development and validation of an immune‐related prognostic signature in lung adenocarcinoma
Source: Cancer Med. 2020 Jun 26;9(16):5960–75. doi: 10.1002/cam4.3240 (PMC7433810; doi:10.1002/cam4.3240)

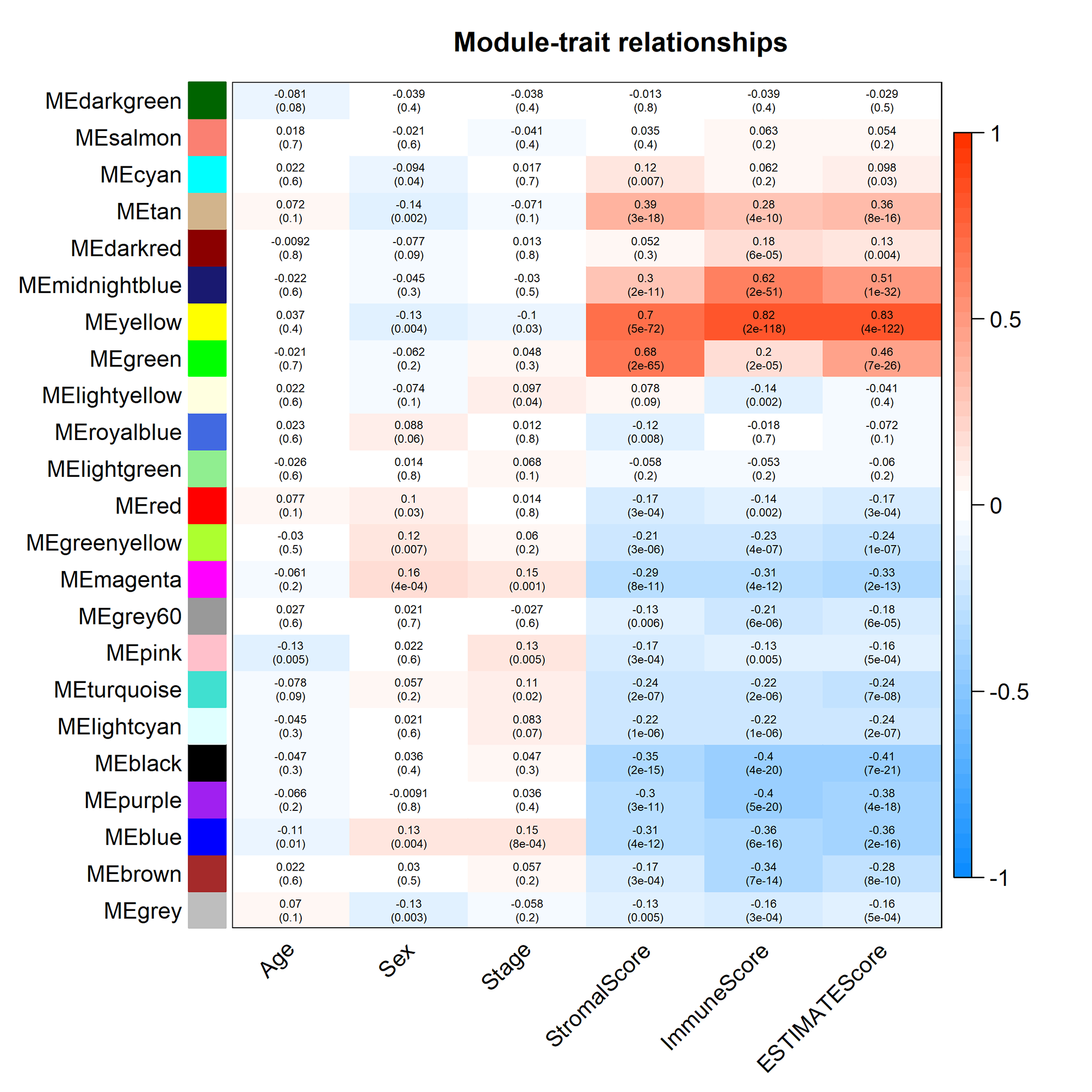

Supplement: Supplementary file 1 — Fig S1 [file CAM4-9-5960-s001.tif]

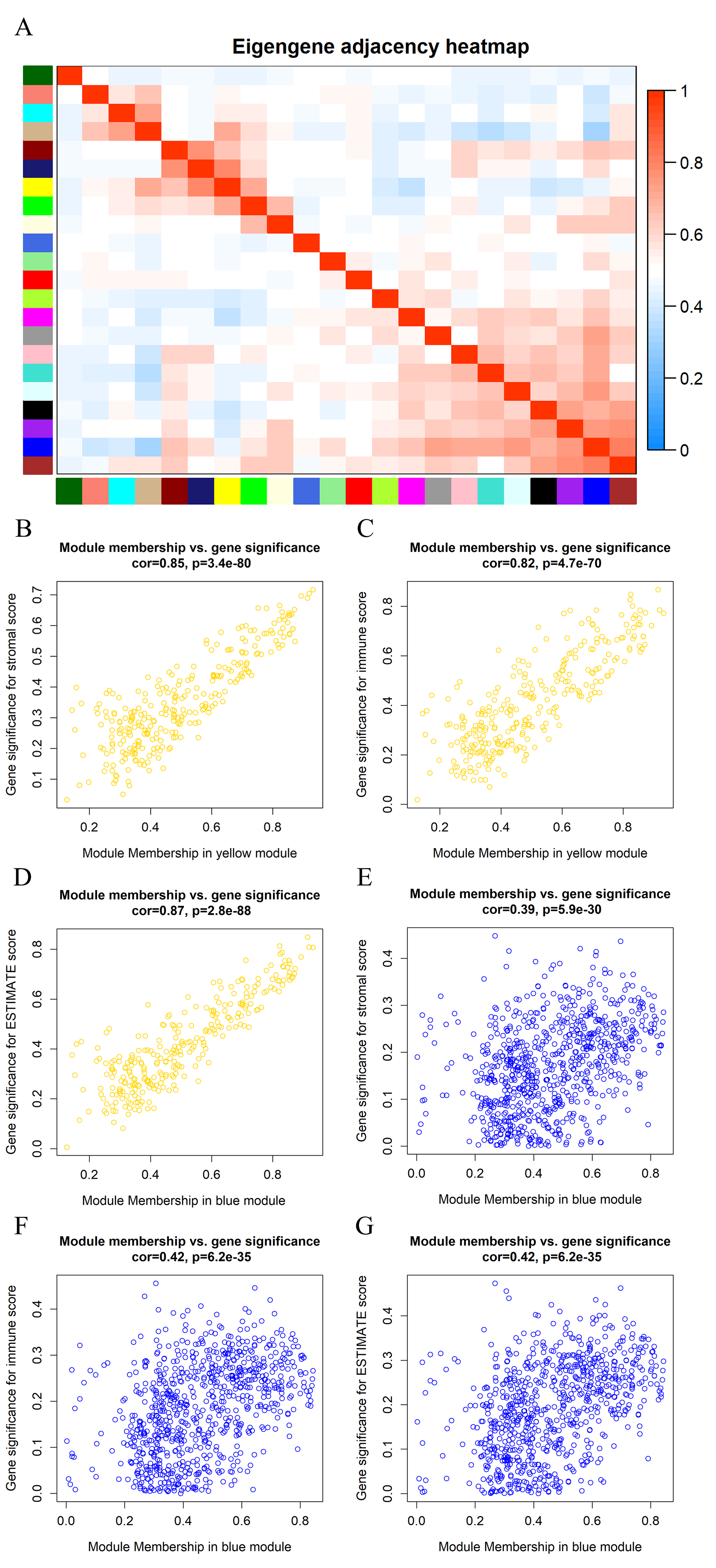

Supplement: Supplementary file 2 — Fig S2 [file CAM4-9-5960-s002.tif]

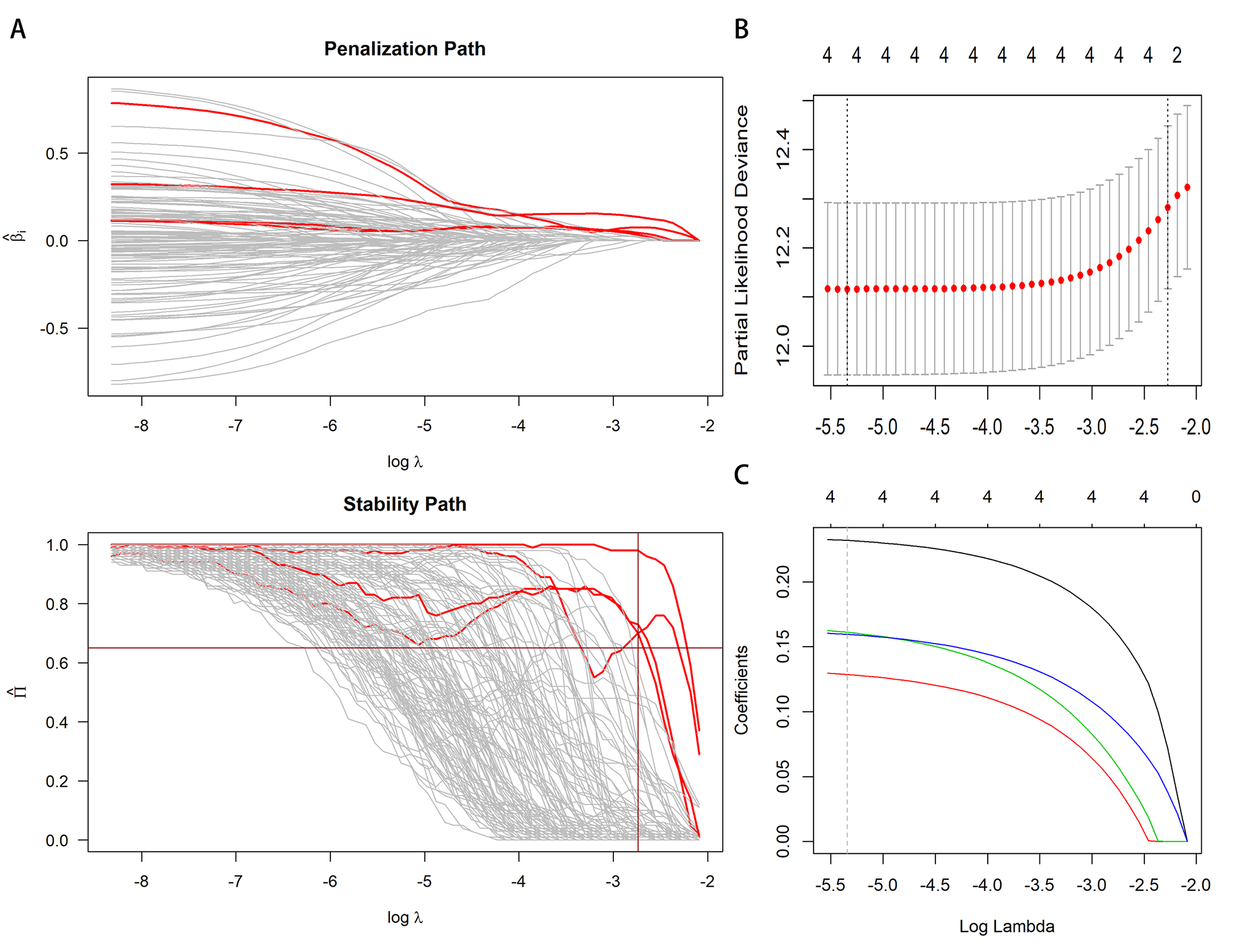

Supplement: Supplementary file 3 — Fig S3 [file CAM4-9-5960-s003.tif]
